# From brain injury to classroom: cognitive and academic outcomes after pediatric stroke. A narrative review

**DOI:** 10.3389/fneur.2025.1680795

**Published:** 2025-10-24

**Authors:** Samuela Tarantino, Martina Proietti Checchi, Michela Ada Noris Ferilli, Gabriele Monte, Alessandro Borrelli, Giuseppe Tiralongo, Massimiliano Valeriani

**Affiliations:** ^1^Developmental Neurology Unit, Bambino Gesù Children’s Hospital, Istituto di Ricovero e Cura a Carattere Scientifico (IRCCS), Rome, Italy; ^2^Academy of Pediatrics, Tor Vergata University of Rome, Rome, Italy; ^3^Systems Medicine Department, Tor Vergata University of Rome, Rome, Italy; ^4^Translational Pain Neuroscience and Precision Medicine, CNAP, Department of Health Science and Technology, School of Medicine, Aalborg University, Aalborg, Denmark

**Keywords:** stroke, children, neuropsychology, school, memory, language, attention

## Abstract

Pediatric stroke represents a rare and clinically significant event, often associated with heterogeneous cognitive sequelae. Early brain injury, particularly during the perinatal period, can result in impaired intellectual functioning and various neuropsychological deficits. Cognitive challenges typically affect language, memory, attention, and executive functions, with their nature and severity influenced by factors such as lesion location, age at onset, and comorbidities like epilepsy or sleep disturbances. Language deficits are commonly observed, particularly in cases involving left-hemispheric or basal ganglia damage, and may endure despite neuroplastic adaptation. Executive dysfunction is also frequently observed, typically involving reduced working memory and cognitive flexibility, and is strongly linked to academic underachievement. Moreover, the diagnosis of secondary ADHD may further complicate the cognitive profile, intensifying challenges related to attention, learning, and behavioral regulation. Despite the high need for tailored educational support, evidence-based cognitive rehabilitation strategies remain limited. Emerging interventions – such as non-invasive brain stimulation and virtual reality – have proven promising, but current evidence is preliminary and lacks validation in youth. Given the elevated risk of long-term academic and functional impairment, early cognitive screening and individualized multidisciplinary intervention are essential to support developmental outcomes in children affected by stroke.

## 1 Introduction

**Figure 1:**
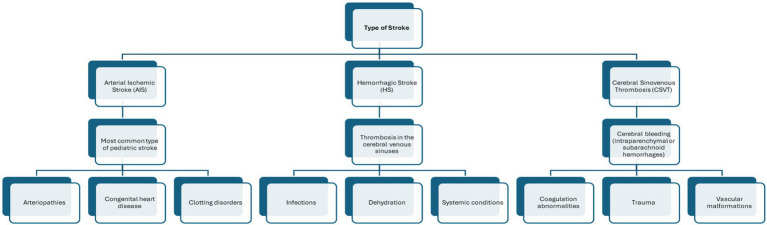
Types of pediatric stroke and associated causes.

Stroke, or cerebrovascular accident, is a sudden neurological event caused by a disruption in cerebral blood flow, leading to brain cell injury or death (1, 2). Pediatric stroke presents distinct challenges due to the ongoing development of the brain. Although its incidence is rare, the consequences of stroke are significant for a child’s development, influencing learning, social skills, and quality of life (3–7). It is classified into three main types: arterial ischemic stroke (AIS), cerebral sinovenous thrombosis (CSVT), and hemorrhagic stroke (HS) (8). AIS is the most common type, often associated with arteriopathies, congenital heart disease, or clotting disorders. CSVT is typically triggered by dehydration, infections, or systemic conditions. HS, including both intraparenchymal and subarachnoid hemorrhages, is usually linked to trauma, vascular malformations, or coagulation abnormalities (8) (Figure 1). According to the timing of onset, pediatric stroke (PS) is classified as perinatal stroke, occurring from the 20th week of gestation to 28 days after birth, and childhood stroke, occurring from 29 days to 18 years of age (9). The incidence of perinatal stroke among live births is approximately 1 in 1,100 (10), while the incidence of childhood stroke ranges from 1.3 to 13 cases per 100,000 children (11).

Among stroke survivors, motor deficits are common, with hemiparesis being prevalent. Data from the literature show that 56–67% of children with ischemic stroke develop hemiplegia, significantly affecting daily activities such as writing, moving, and participating in physical education (12). Motor deficits affect 89% of children after stroke, with 40% requiring special education services (13). Unilateral cerebral palsy or hemiplegia may be a common adverse motor outcome in children with perinatal ischemic stroke, potentially affecting up to 84.9% of those with motor impairments (12).

Acute neurological events during critical developmental periods can cause persistent cognitive, emotional, and social difficulties. Children affected by traumatic brain injury or pediatric brain tumors often experience impairments in attention, memory, learning, and executive function. These deficits negatively impact academic performance and peer relationships, often requiring tailored support (14–18). The physical consequences of pediatric stroke, such as motor impairments, are well established (12, 13, 19). However, its long-term impact in the educational context—particularly on cognitive functioning, academic achievement, school adaptation, and peer integration—remains insufficiently investigated. Survivors often experience deficits in language, attention, memory, and executive processes. These difficulties can significantly affect their ability to meet the cognitive demands of the school environment (4, 20–22). As a result, stroke survivors may struggle to keep up with their peers in school, leading to academic delays and difficulties in meeting educational milestones. Long-term outcomes are influenced by multiple factors, including the age at stroke, lesion location and extent, neuroplasticity, and the availability of appropriate rehabilitative and educational support (4, 22). However, the existing data are not conclusive, with findings varying across studies.

The school environment plays a crucial role in addressing both academic and emotional needs. It may exacerbate the emotional and interpersonal challenges faced by pediatric stroke survivors, thereby increasing their susceptibility to social isolation and academic stress as a result of difficulties in peer interactions, emotional regulation, and adaptation to classroom dynamics (22, 23). Nonetheless, the school setting also constitutes a vital context for support and developmental opportunities (22, 24).

Despite increasing awareness of the challenges faced by pediatric stroke survivors, research on their adaptation to school life remains scarce. Existing reviews have primarily addressed general cognitive functioning or rehabilitation strategies, with limited focus on school-related performance and educational outcomes (6, 14, 25–29).

Existing research on pediatric stroke provides fragmented insights into its impact on academic performance and learning progression. Moreover, the contributions of cognitive rehabilitation and school-based interventions are still being delineated, leading to a limited understanding of educational and cognitive outcomes in affected children.

### 1.1 Aims

This review has two main aims: (1) to provide a comprehensive synthesis of the impact of pediatric stroke on school-related outcomes. For this purpose, we will examine how stroke affects core cognitive domains, including attention, language, and memory, and the consequent effects on academic performance and learning progression; (2) to explore cognitive rehabilitation approaches and school-based strategies that may support children’s academic achievement and successful integration into the school environment.

## 2 Method

### 2.1 Search strategy

This is a narrative review of the literature on pediatric stroke. A narrative approach was chosen due to the substantial heterogeneity of available studies, which differ in design, participant characteristics, stroke types, ages at onset, and cognitive and academic outcome measures. The included studies span case reports, longitudinal cohorts, and cross-sectional investigations, precluding direct comparison or quantitative synthesis. This approach allowed a comprehensive integration of findings across multiple domains, including cognitive sequelae, executive functions, academic performance, rehabilitation strategies, and school reintegration. Relationships and patterns across studies were explored, and gaps in the literature were identified. Narrative synthesis enabled the identification of overarching patterns and the examination of gaps within the existing evidence base. A comprehensive literature search was conducted across major academic databases, specifically PubMed and Scopus, covering studies published between 2000 and 2025.

The search strategy included combinations of the following keywords:

- Population: “pediatric stroke,” “neonatal stroke”
- Cognitive outcomes: “cognitive outcomes,” “cognitive deficits,” “language problems,” “memory problems,” “attention problems,” “executive difficulties,” “visuo-spatial difficulties”
- Academic/functional outcomes: “school problems,” “academic difficulties,” “school integration,” “cognitive rehabilitation”

An example of a search string used:

(“pediatric stroke” OR “neonatal stroke”) AND (“cognitive outcomes” OR “cognitive deficits” OR “language problems” OR “memory problems” OR “attention problems” OR “executive function difficulties” OR “visuo-spatial difficulties”) AND (“school problems” OR “academic difficulties” OR “school integration” OR “cognitive rehabilitation”).

Reference lists of all included studies and relevant review articles were also manually screened to identify additional eligible publications.

### 2.2 Inclusion and exclusion criteria

Inclusion criteria:

- Children and adolescents (0–18 years) with a history of pediatric or neonatal stroke
- Studies reporting cognitive or academic outcomes
- Longitudinal, cross-sectional, or interventional designs

Exclusion criteria:

- Studies focusing on adults (>18 years)
- Studies on unrelated neurological conditions
- Non-full-length publications (e.g., abstracts, editorials)

### 2.3 Study selection

**Figure 2:**
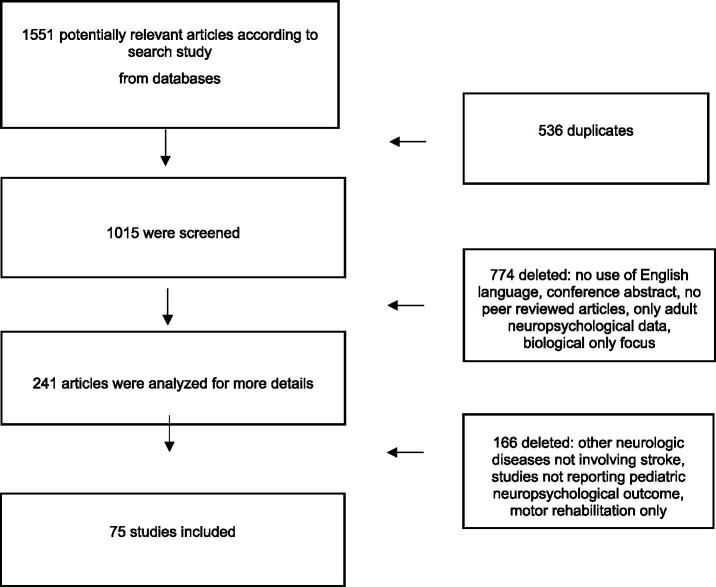
Flow diagram of the study methodology.

The selection process involved two stages:

- Phase 1 – Title and abstract screening: Two independent reviewers (S. T. and M. P. C.) screened titles, abstracts, and keywords to identify potentially relevant studies. Studies with insufficient abstract information were retained for full-text review. Duplicate articles were removed. Studies that did not address cognitive or academic outcomes related to pediatric or neonatal stroke were excluded.
- Phase 2 – Full-text screening: Four additional reviewers (G. M., M. A. N. F., A. B. and G. T.) independently assessed full texts for eligibility according to the inclusion and exclusion criteria detailed in section 2.2. Disagreements were resolved through consensus discussion, supervised by the senior author (M. V.). Main reasons for exclusion at the full-text stage were recorded and are presented in Figure 2.

### 2.4 Data extraction and synthesis

Data were extracted for each included study, including:

- Participant characteristics (age at stroke, stroke type, sample size, control group)
- Cognitive and academic outcomes
- Evaluation methods and study procedures

The narrative synthesis involved the following steps:

1. Preliminary synthesis: organizing studies according to cognitive domains (attention, memory, executive function), academic performance, and school integration challenges.
2. Exploring relationships: examining studies collectively to identify common trends, consistent findings, and interactions between cognitive and academic outcomes.
3. Assessing the synthesis: key findings were described in a qualitative and narrative manner, with attention to their relevance in relation to the review’s objectives. A basic assessment of study quality was carried out, considering general aspects such as study design, sample size, and methodological clarity. This allowed contextualization of the strength of the evidence and identification of potential limitations within the literature.
  Figure 2 illustrates the study selection process.

## 3 Results

### 3.1 Cognitive sequelae

Cognitive outcomes following pediatric stroke vary widely, from typical development to severe impairments. These differences are influenced by several factors (20, 30–33). A critical determinant of cognitive outcomes is the timing of the stroke, which plays a pivotal role in shaping long-term cognitive function (6, 34). Some studies show that strokes occurring before the age of 1 are associated with poorer cognitive outcomes (35, 36), while others suggest that strokes occurring later in childhood, particularly AIS when brain maturation is more advanced, result in worse cognitive outcomes (30). In a previous review, Rees et al. reported that children with perinatal stroke had IQ scores more than 24 points lower than their peers, with deficits becoming more pronounced as academic demands increased. The authors also noted substantial heterogeneity across studies in terms of assessment timing, assessment tools, and the inclusion of both left- and right-sided strokes (6). In an earlier investigation, Westmacott et al. (37) observed that, although children with unilateral neonatal strokes show average IQ scores during preschool, these tend to decline during the school years. In a later study, the authors found that stroke timing influences cognitive outcomes. Patients with perinatal strokes had the lowest mean IQ (91.63), followed by those who sustained a stroke between 1 month and 5 years (95.42), and those between 6 and 16 years (97.21) (35) (Supplementary Table S1). The authors reported that subcortical strokes had the most substantial impact on intellectual ability and information processing skills when they occurred during the prenatal or perinatal period. Cortical strokes in the same period were less frequently associated with cognitive deficits. Children who experienced cortical strokes between 1 month and 5 years of age showed lower performance across multiple cognitive domains compared to those with cortical strokes occurring either earlier or later in development (35).

These results highlight the important roles of both timing and lesion location in cognitive outcomes. Supporting this view, Anderson et al. found that children with larger or more extensive lesions, particularly those involving both cortical and subcortical regions, have lower IQ scores, whereas a better acute neurological status and the absence of seizures predict higher IQ outcomes (33). These findings underscore the importance of early neurological health and lesion characteristics in shaping long-term cognitive outcomes such as IQ.

In addition to stroke timing and location, motor recovery may play a role in cognitive function. Ledochowski et al. (38) reported that children who exhibited better motor recovery in the first year following the stroke have higher IQ scores, particularly in domains such as verbal ability, visuo-perception, and processing speed. However, cognitive function, more than motor recovery, is a stronger predictor of long-term educational needs. This highlights the importance of addressing cognitive challenges during rehabilitation, as these deficits often have a more lasting impact than motor impairments (13).

The presence of comorbidities, particularly post-stroke epilepsy, can further complicate the cognitive outcome. In this regard, children who experience stroke during mid-childhood (ages 5–10 years) have the most favorable prognosis, whereas those with stroke before the age of five or after the age of 10 have less favorable cognitive outcomes (30). Neonates, especially those with epilepsy, have poor cognitive outcomes, underscoring the significant role that comorbidities, such as epilepsy, play in the overall prognosis of cognitive function following pediatric stroke. Gschaidmeier et al. (39) found that children with post-stroke epilepsy exhibit significantly lower non-verbal IQ scores, particularly in abstract reasoning and visuospatial tasks, compared to their peers without epilepsy. These findings are consistent with earlier studies demonstrating progressive cognitive impairment linked to post-neonatal epilepsy in children following perinatal AIS (40).

Extending the focus from cognitive outcomes, language abilities represent another critical aspect of post-stroke development. Language impairments, frequently observed following stroke, can significantly disrupt both expressive and receptive communication, compromising the individual’s ability to understand verbal instructions and participate in social interactions (41–43). Children who experience a stroke during childhood often face considerable challenges in language development, with the timing, location, and type of stroke playing pivotal roles in determining the extent of these impairments (44–51).

The perinatal period, in particular, represents a window of heightened vulnerability and opportunity in brain development. Some studies have found that children who suffered a stroke before the age of 1 exhibit significantly poorer outcomes compared to those who experience a stroke later in life (44, 45). In a study involving 15 patients and their healthy sibling, Newport et al. showed that perinatal strokes, especially those affecting the left hemisphere, can lead to long-term atypical lateralization of language functions, with compensatory shifts to the right hemisphere (46). However, this reorganization does not always ensure full recovery (47). In a previous study, François et al. reported the case of a 3.5-year-old child with a left-sided perinatal stroke (49). Despite undergoing extensive brain reorganization to develop language, the child did not achieve full functional recovery. In contrast, children with right hemisphere lesions perform comparably to controls, though they show reduced use of complex syntax (47).

Peterson et al. investigated the impact of childhood strokes involving the basal ganglia on language and academic outcomes (50). They found that strokes affecting the left basal ganglia are associated with higher-order language difficulties in verbal fluency, narrative, and pragmatic language. Involvement of these areas leads to an increased risk of academic challenges, including learning disorders.

In addition to lesion location, the type of stroke plays a significant role in determining language outcome. Sherman et al. explored the incidence of language impairments in children with AIS or cerebral venous sinus thrombosis (CSVT) (51). Around 48.7% of children with AIS show initial language impairment, with a persisting delay in 74% of patients with neonatal ischemic stroke (Supplementary Table S1).

Despite the paucity of studies on environmental factors, emerging evidence suggests that bilingual exposure may influence recovery following pediatric stroke. Leung et al. reported comparable overall cognitive outcomes between monolingual and bilingual children; however, bilinguals who experienced stroke within the first year of life, exhibited superior productive language outcomes compared to monolingual peers (52). These findings suggest that bilingual environments may support post-stroke language recovery, with experiential factors influencing outcomes alongside lesion timing and location.

Extending the examination of post-stroke cognitive sequelae, memory constitutes a critical domain frequently affected in pediatric stroke. Impairments may encompass working, verbal, and episodic memory, reflecting the vulnerability of these systems during early neurodevelopment and their susceptibility to disruption following cerebral injury (7, 53–56).

Kolk et al. (7) investigated how strokes occurring very early in life, such as during the neonatal period, can lead to severe memory impairments. In their study, 21 children with neonatal strokes (mean age 6.86 years) and 10 children with strokes later in childhood (mean age 8.21 years) were assessed. The neonatal stroke group exhibited the most significant impairments, particularly in the visuospatial domain, which was more affected than in the childhood stroke group. Particularly in the neonatal stroke group, memory difficulties were pronounced in sentence repetition tasks, suggesting a disruption in the development of verbal memory and phonological processing. However, a study by Abgottspon et al. (53) offers a more nuanced perspective, revealing a nonlinear, U-shaped association between age at stroke and long-term memory outcome. Indeed, children with stroke during early childhood (between 29 days and under 6 years of age) exhibited more pronounced memory deficits than those with neonatal or later-onset strokes.

Also, the location and lateralization of the lesion play a crucial role in shaping memory outcome (7). In a cross-sectional study involving 32 children (aged 6–14 years), Fuentes et al. (55) found that left hemisphere strokes, particularly those affecting the middle cerebral artery, are strongly associated with deficits in working memory. Episodic memory, which is vital for recalling personal experiences and organizing narrative information, is often disrupted when strokes affect medial temporal lobe structures. Gold and Trauner (56) showed that perinatal strokes leading to reduced hippocampal volume impair a child’s ability to retain and organize narrative details, such as recalling stories, events, and sequences. Reduced hippocampal volumes are associated with impaired memory performance, with left-sided reductions predominantly affecting verbal memory and right-sided reductions impacting non-verbal memory. Furthermore, the occurrence of seizures contributes to both memory deficits and additional hippocampal volume loss. A recent study by Salzmann et al. (57) provides novel longitudinal data indicating that lesion volume and involvement of basal ganglia structures, particularly the left caudate nucleus, predict long-term working memory and processing speed after childhood stroke. Neurological function at discharge and follow-up was also found to be an important predictor. This study advances previous research by combining quantitative lesion metrics with repeated neurological assessments, although its focus was limited to selected cognitive domains and sample size was relatively small.

Deficits in executive functions (EF) are common in children with a history of stroke, independent of intellectual abilities (7, 58–65). Strokes occurring during the perinatal or early childhood period may disrupt EF maturation by damaging brain networks critical for cognitive control and self-regulation, such as the frontoparietal and frontostriatal systems (62). These impairments often hinder the child’s ability to follow routines and cope with increasing cognitive demands, especially in structured school settings. (25). Morphometric and behavioral studies show that children with AIS often exhibit elevated parent-rated ADHD symptoms and EF impairments, including deficits in working memory, planning, and organization. In contrast, children with periventricular venous infarction (PVI) tend to show fewer deficits, highlighting the role of lesion type in cognitive outcomes. Lesion size also predicts long-term cognitive outcomes, such as processing speed and EF, with larger lesions linked to poorer performance (66). According to studies by Rivella et al. (5) and Rivella and Viterbori (25), EF impairments caused by perinatal or pediatric stroke are more severe when the lesion is large and leads to language deficits. Li et al. (63) suggested that EF abnormalities could represent a key predictor of learning difficulties, particularly in mathematics.

Functional neuroimaging studies demonstrate that lesions during sensitive developmental periods cause lasting changes in neural connectivity. Larsen et al. reported that reduced interhemispheric frontal connectivity correlates with poorer attention and executive performance in children with perinatal stroke (62). Atypical resting-state activity in the default mode network also associates with deficits in cognitive flexibility and processing speed (64). In contrast, Kolk et al. (7) observed that EF often remains spared in children who experienced a stroke during the neonatal or early childhood period, despite impairments in other cognitive domains, such as attention and memory.

Brain network disruptions following pediatric stroke contribute to attentional difficulties and increase the risk of secondary ADHD (S-ADHD), affecting 13.1% of post-stroke children (67, 68). Children with neonatal AIS display persistent attention deficits into adolescence, suggesting long-term executive control alterations (68).

Neuropsychological vulnerabilities in attention and EF are independent of the involved hemisphere (68). Long et al. (60) demonstrated that pediatric brain lesions, irrespective of location, can impair executive functions, including attention, cognitive flexibility, goal-setting, and information processing. Steinlin reported a child with cerebellar infarction who exhibited severe attentional difficulties similar to those seen in attention deficit disorders. This finding underscores the cerebellum’s important role in attention regulation (69). The severity of the dysfunction is strictly related to the size of the lesion, at least regarding attentional capacity (60, 70).

Pediatric stroke can lead to visuospatial impairments, with visual neglect and deficits in spatial processing frequently observed, particularly following right-hemisphere lesions.

Purpura et al. (71) found that although visual neglect is less common in children than in adults, neglect-like behaviors are observed, especially in patients with right hemisphere lesions, particularly during the perinatal period. These deficits can significantly impact daily functioning and academic performance, although further research is needed to fully understand their mechanisms. Children who experience stroke also face challenges in spatial processing, affecting both motor and cognitive functions. Everts et al. (72) reported that right hemisphere lesions, particularly those occurring early in life, are associated with deficits in spatial attention and neglect-like symptoms. Left hemisphere lesions, in contrast, are primarily linked to language and verbal memory deficits (72). A recent study by Nenning et al. (73) provides important integration with existing data, highlighting how spatio-temporal alterations in brain connectivity may underlie the neural mechanisms responsible for visuo-spatial difficulties observed in children with AIS.

Cognitive deficits may directly impact school learning. They impair the acquisition, consolidation, and application of academic skills, including reading, writing, and mathematics (50, 59, 63, 74–76). A growing body of research has highlighted the substantial impact of pediatric stroke on children’s educational outcomes. In a follow-up study, 64% of children demonstrated mild to severe impairments in school-related activities and academic performance (74). EF, including working memory, planning, and attention, are critical for mathematical learning. Damage to these functions, which is frequently observed following stroke, can lead to dyscalculia. This condition is characterized by difficulties in numerical understanding and calculation (63).

Secondary ADHD is another complication commonly observed in pediatric stroke survivors. It exacerbates academic difficulties by disrupting attention regulation, organizational skills, and task completion. This condition exacerbates academic difficulties by disrupting attention regulation, organizational skills, and task completion. In a longitudinal study, Roberts et al. followed children with secondary ADHD (S-ADHD), stroke-only, and developmental ADHD (D-ADHD) for about 4 years to assess academic outcomes (59). They found that S-ADHD children experience greater worsening in reading, while math scores decline similarly across groups. Both ADHD groups show high rates of learning disabilities. No individual or neurological factors predict academic decline, but ADHD symptoms are linked to poorer sustained attention and organizational skills.

Metacognition, the ability to monitor and regulate one’s own cognitive processes, also plays a vital role in academic success. Stroke-related disruptions in metacognitive skills can hamper the use of effective learning strategies, limiting both reading comprehension and mathematical problem-solving. Deotto et al. (76) reported that 40% of children with stroke exhibit clinically significant impairment in pencil-and-paper arithmetic, primarily due to deficits in planning, monitoring, and cognitive regulation.

Damage to specific brain regions, such as the basal ganglia, can further impair academic performance by disrupting phonological processing, a crucial component of reading and writing. This damage may also contribute to learning difficulties, including dyslexia. Peterson et al. (50) found that children with stroke-related damage to the basal ganglia often struggle with word decoding, severely impacting literacy skills. Mathematical abilities are also affected, suggesting that basal ganglia damage has broad implications across academic domains.

### 3.2 Rehabilitation and educational support

Cognitive rehabilitation plays a key role in the recovery of children after neonatal or pediatric stroke. It addresses deficits in attention, memory, executive functions, and other domains essential for learning and academic success.

Non-pharmacological interventions, including cognitive enhancement programs and neuropsychological therapies, are central to restoring these cognitive functions. Despite their widespread use, the efficacy of traditional rehabilitation approaches in pediatric stroke is still supported by limited evidence (4, 26).

Direct neuropsychological rehabilitation approaches in children are usually divided into two categories: substitution and restoration. Substitution involves teaching alternative strategies or modifying environments to compensate for cognitive deficits, leveraging the child’s strengths to minimize impairment. Restoration, instead, relies on targeted exercises to improve impaired cognitive abilities, particularly attention and executive function (77). Most studies on non-pharmacological interventions in pediatric stroke have primarily focused on motor rehabilitation, while cognitive outcomes remain relatively understudied (26). Mrakotsky et al. (78) highlighted that, although interventions addressing sensorimotor and speech/language deficits are more advanced, those targeting higher-order cognitive and behavioral impairments are still underdeveloped. They advocate for pediatric-specific, multidisciplinary approaches rather than adaptations of adult rehabilitation models (78).

Speech-language therapy primarily targets motor speech and basic language deficits. However, in early childhood stroke, children often develop higher-order language impairments, such as difficulties with discourse processing, verbal fluency, and organizing ideas, which are rarely addressed by standard interventions (50, 79). Case reports suggest that speech-language therapy, when integrated with other interventions, may offer additional benefits, even though empirical evidence in pediatric stroke is still limited (80, 81).

Controlled trials in cognitive rehabilitation are mostly derived from adult stroke or pediatric acquired brain injury populations and often target single domains (e.g., working memory or motor skills), with limited generalization to daily life or academic performance (79). Early research investigated the effects of combining memory training with academic tutoring in children with sickle cell disease and stroke (SCD). Yerys et al. (82) conducted a 6-week pilot study involving six children with SCD-related cerebral infarcts. Participants were divided into two groups: one group (*n* = 3) received standard tutoring alone, while the other group (*n* = 3) received tutoring supplemented with adjunctive training in memory strategies, specifically silent rehearsal and semantic organization. The group receiving the combined strategy training demonstrated significantly greater improvements in memory performance compared to the tutoring-only group (82). Few years later, King et al. (83) conducted a 2-year educational rehabilitation study in a very small sample (11 children) with SCD and cerebral infarcts, specifically targeting memory deficits. Children who received general tutoring combined with targeted memory strategy training showed greater improvements in verbal memory and backward Digit Span compared to those who received tutoring alone. However, these cognitive gains did not clearly lead to significant improvements in academic achievement (reading, math, and spelling). Academic performance remained modest and similar across both groups, highlighting a gap between improved memory and real-world academic outcomes. Gilardone et al. (81) reported a case of a 13-year-old with AIS, in whom an intensive, individualized rehabilitation program yielded cognitive and functional improvements, partly sustained at follow-up. In another study, Eve et al. (84), using Cogmed, a computerized program designed to train working memory through specific exercises aimed at enhancing cognitive skills related to learning, showed short-term working memory improvements but no sustained academic benefit after 12 months.

Technological innovations offer promising adjuncts to traditional methods, activating neuroplastic pathways through repetitive, individualized practice (27, 80). Virtual reality-based rehabilitation, primarily validated in adults, is potentially effective in improving attention, memory, and EF, with benefits in cognition, motor function, and balance (27). Evidence for rTMS in pediatric AIS is extremely limited and restricted to isolated case reports. Carlson et al. (80) described a 15-year-old patient with post-stroke expressive dysphasia who demonstrated language improvements following inhibitory rTMS to the contralesional right inferior frontal gyrus combined with intensive speech therapy. Although well tolerated and partly sustained at follow-up, this intervention remains experimental. Caution is necessary due to potential risks, including a reduction in seizure threshold, which may increase seizure risk, particularly in patients with epilepsy or other neurological vulnerabilities (85).

Rehabilitation following pediatric stroke should not only target cognitive recovery but also support the child’s reintegration into everyday life, including academic, social, and functional domains. In this context, school-based interventions play a key role in facilitating meaningful participation and long-term adjustment.

Approximately 40% of children with pediatric ischemic stroke require special education services, and 19% attend specialized schools (22). Hawks et al. (86) reported the educational placements of children who experienced a pediatric intracerebral hemorrhage. Among these children, 46.7% attended age-appropriate regular classes, 40% received in-class support, 10% were enrolled in special education, and 3.3% required home-based services. The type of educational placement appears to influence recovery trajectories significantly. Indeed, children who receive in-class support have better academic and social integration compared to those placed in separate special education settings. Cognitive factors play a central role in educational outcomes. Yvon et al. (13) reported that cognitive deficits, particularly in IQ, are stronger predictors of academic performance than motor impairments. These findings emphasize the need for educational interventions tailored to each child’s specific neurocognitive profile (13).

Several school-based educational programs have proven promising. Proios et al. (24) demonstrated the effectiveness of a school-based educational program in Greece aimed at improving stroke awareness among students. Williams et al. (87) developed the “Hip Hop Stroke” program. This initiative educates elementary school children in high-risk communities on recognizing stroke symptoms.

Leib et al. (88) implemented a quality improvement initiative to support school reintegration for children hospitalized with acute neurological conditions. They established a neuropsychology consult workflow with school reintegration recommendations and staff training. In 12 months, 36 consults were completed, with recommendations increasing from 0 to 100%. Patients included those with stroke, neuroimmune disorders, cardiac arrest, TBI, encephalitis, and brain tumors. The initiative was feasible and practical, but further research is needed to evaluate outcomes and sustainability.

Although the role of teachers in the recovery of children after pediatric stroke is essential, it has been insufficiently explored in the existing literature. While cognitive rehabilitation is central to recovery, teacher involvement is equally important for academic and social integration. Emerging evidence emphasizes the need for adequate teacher preparation to address the educational challenges of pediatric stroke (22). Vanderlind et al. (22) highlighted a gap in teacher training. Teachers may struggle to provide necessary support without adequate resources and knowledge. McKevitt et al. (89) reported that parents often act as intermediaries between the health and education systems. They advocate for their child’s needs while navigating fragmented support structures (89). This approach promotes better communication, consistent care, and more responsive educational planning.

## 4 Discussion

This review synthesizes the existing literature on the impact of pediatric stroke on school-related outcomes, a topic less explored compared to broader cognitive sequelae and rehabilitation. The evidence highlights the complex relationship between neuropsychological impairments and academic performance, with particular attention to the challenges faced during school reintegration after pediatric stroke. Pediatric stroke disrupts the developmental trajectory of children, resulting in long-term cognitive, emotional, and academic consequences. Although typically viewed as a medical issue, growing evidence shows that pediatric stroke impacts not only physical health but also cognition, emotional well-being, and broader aspects of school functioning. Rehabilitation can enhance neurological function. However, these improvements do not consistently translate into academic achievement. This suggests that gains observed in clinical settings may not fully meet the complex demands of the educational environment. Pediatric stroke is frequently associated with impairments in executive functioning, attention, working memory, and language. These deficits interfere with essential processes for academic success and often lead to significant difficulties in school (7, 53, 58, 59, 63, 90). For example, executive dysfunction impairs goal-directed behavior and cognitive flexibility, limiting the child’s ability to initiate, organize, and adaptively regulate problem-solving strategies. These challenges are often compounded by attentional deficits. Such deficits reduce the capacity for sustained focus and task persistence, ultimately hindering effective engagement in academic activities (60–62, 68, 76). Furthermore, limitations in working memory, critical for the transient storage and manipulation of information, disrupt the efficient encoding and retrieval of new material, thereby affecting higher-order learning and the acquisition of complex skills (53, 55). Language impairments can also hinder both expressive and receptive communication, impacting the child’s ability to follow verbal instructions and interact with peers (42, 43). Motor impairments primarily affect physical function. However, they may also complicate academic reintegration by interfering with cognitive and social processing (39, 91).

The consequences of pediatric stroke are influenced by factors such as age at stroke onset, lesion location, and comorbidities like epilepsy (92–94). The relationship between age at stroke onset and recovery outcome is complex and subject to debate. The developing brain has significant neuroplastic potential. This potential enables functional recovery, especially during critical periods of neural maturation (43, 44, 95, 96). During these windows, plasticity facilitates the reorganization of neural circuits, allowing alternative pathways to compensate for functional impairments. This adaptive capacity, however, has limitations. Maladaptive plasticity, characterized by inefficient or atypical reorganization, may hamper recovery and contribute to atypical maturation of the neural systems (4, 43). Two main theoretical frameworks address this dual role. The early plasticity hypothesis posits that the immature brain has an enhanced capacity for compensatory reorganization. The early vulnerability hypothesis suggests that early injuries may disrupt critical neurodevelopmental processes, leading to long-term impairments (43, 95, 96). Empirical findings support both perspectives, revealing a non-linear pattern of recovery. For example, strokes occurring between 1 month and 6 years of age are frequently associated with poorer cognitive outcomes compared to those occurring in neonates or later in childhood (53). This pattern may reflect the heightened vulnerability of specific cognitive and neural systems during sensitive developmental windows.

School should not be viewed solely as a place where difficulties arise. It also serves as a therapeutic environment that actively supports recovery Schools can foster cognitive and emotional development by offering structured activities. They can also provide personalized learning strategies and opportunities for social engagement (22, 24, 29, 87). Effective educational support includes individualized instruction, teacher training, and coordinated efforts between educators and healthcare professionals (22). Developing Individualized Education Plans (IEPs) tailored to each child’s cognitive, emotional, and behavioral needs can significantly enhance academic performance. These plans may include adaptive strategies, extra time for tasks, and assistive technologies (4, 97). Moreover, promoting positive peer interactions and combating social isolation through inclusive programs can strengthen well-being and resilience (22, 24, 87). With the right strategies and collaboration, schools can turn educational challenges into valuable opportunities for growth.

This review comprehensively integrates heterogeneous literature on pediatric stroke outcomes. It elucidates how lesion features, timing, neurological recovery, and comorbidities collectively shape cognitive and academic development. A key strength of this review is its focus on real-world academic outcomes, bridging the gap between neuropsychological sequelae and school performance-a perspective largely underrepresented in previous literature (7, 22, 53, 63). Furthermore, by integrating heterogeneous studies across multiple cognitive domains and stroke types, the review offers a broad understanding of potential recovery trajectories and educational challenges.

The findings of the present review have practical relevance for clinicians and educators supporting children after pediatric stroke. Early, comprehensive assessment of cognitive, language, and executive functions is essential to identify children at risk and guide individualized interventions, including IEPs and rehabilitation strategies. Schools play a pivotal role by implementing personalized learning strategies, adaptive tools, and social support, while teacher training can enhance inclusion and reduce barriers to learning. Finally, integrating multidisciplinary approaches that combine neurological, cognitive, and educational strategies can optimize academic and overall outcomes, turning schools into therapeutic environments that complement medical care.

### 4.1 Limitations of the review

Despite these strengths, several methodological limitations warrant consideration. The included studies show substantial heterogeneity. This includes age at stroke onset, lesion characteristics, stroke subtypes, comorbidities, assessment instruments, and timing of evaluations. Such variability complicates direct comparisons and limits the generalizability of findings (53, 55). Such variability may partly explain some inconsistencies in the cognitive outcomes reported. In this contest, Kolk et al. (7) included only children with an IQ of 80 or above, which could account for their observation that executive functions were often spared. These results diverge from the observations reported by Larsen et al. included children with a broader range of cognitive abilities, capturing more pronounced executive deficits (62). This underscores how variations in inclusion criteria, sample characteristics, and assessment methods can influence reported outcomes. Many studies are limited by small sample sizes and the absence of control groups, which reduces the reliability and generalizability of the reported outcomes and interventions (Supplementary Table S1). Most included studies originate from Western, high-income countries and are published in English, potentially introducing language and publication biases that limit the generalizability of findings to non-Western populations. The influence of cultural and socioeconomic factors on psychological development, rehabilitation, and school integration following pediatric stroke remains insufficiently explored. Yet, extensive evidence shows that socioeconomic status (SES) significantly impacts cognitive development and access to educational resources. Children from disadvantaged backgrounds tend to perform worse in executive functions, language, and memory than their more advantaged peers (98, 99). These disparities likely affect both rehabilitation outcomes and school reintegration but remain under-addressed in pediatric stroke research. Beyond SES, family environment and cultural dynamics also shape social participation and access to rehabilitation (100, 101). Qualitative studies indicate that cultural values, such as family responsibility, can either support or hinder engagement with post-stroke rehabilitation programs. A qualitative study on social participation among stroke survivors further highlighted how cultural norms, including filial piety, influence social engagement and indirectly limit access to rehabilitation (100). Although focused on adults, these findings offer valuable insight into the sociocultural factors that shape recovery. They underscore the need for culturally sensitive interventions that consider family and community values to improve participation outcomes.

Future studies should systematically incorporate cultural and socioeconomic variables and perform rigorous bias assessments to enhance the applicability and validity of findings.

Another gap concerns the limited integration of medical, neurocognitive, and educational perspectives. Although cognitive and academic outcomes are often reported, few studies assess how rehabilitation strategies translate into meaningful improvements in school functioning or incorporate multidisciplinary approaches (22, 24, 87). Future research should investigate how coordinated interventions—combining neurological, cognitive, and educational strategies—can optimize long-term outcomes and support children’s academic achievement.

## 5 Conclusion

Pediatric stroke represents a complex neurological event that profoundly affects cognitive and functional abilities. Effective rehabilitation is crucial to support recovery and optimize outcomes for affected children. The school setting must be recognized as a vital component of the rehabilitation process, providing an environment where cognitive and functional skills can be reinforced and applied in real-life contexts. Tailored educational interventions, coordinated with medical and rehabilitation teams, enhance the child’s reintegration into academic life and support ongoing recovery. Multidisciplinary collaboration among healthcare providers, therapists, educators, and families is essential to deliver comprehensive care and maximize the potential for successful rehabilitation and academic achievement in pediatric stroke survivors.
