# Supplementary material for: From brain injury to classroom: cognitive and academic outcomes after pediatric stroke. A narrative review
Source: Front Neurol. 2025 Oct 24;16:1680795. doi: 10.3389/fneur.2025.1680795 (PMC12591957; doi:10.3389/fneur.2025.1680795)
Supplement: Supplementary file 1 [file Table_1.pdf]

Table 1. Summary of the results from the available cognitive studies.

Author, Year	Sample Characteristics	Age at Stroke Onset	Cognitive Functions Assessed	Follow-up / Timing	Main Findings / Results	Limitations of the studies
Ganesan V et al., 2000 (34)	N: 128 patients<br>Type: AIS (excluding neonatal stroke)<br>Age: 6 months – 29 years<br>Controls: none	3 months- 15 years	IQ, language, activities of daily living, motor, speech, behavioral, and educational domains	Median 3 years post-stroke (range 3 months- 13 years)	The majority of children exhibited significant impairment, with younger age predicting poorer outcomes. Parental reports correlated well with therapist assessments but less consistently with neuropsychological evaluations	Lack of control group<br>Small subgroup with formal assessments<br>Short follow-up duration<br>No standardized behavioral scale used<br>Subjective parental reporting
Westmacott R et al., 2010 (35)	N: 145 patients<br>Type: Unilateral AIS (lesion location: subcortical, cortical, combined)<br>Age: 3.5 – 12.4 years<br>Controls: none	Perinatal to 16 years (3 groups: perinatal, 1 month-5 years, 6-16 years)	IQ, verbal ability, working memory, processing speed	Mean 1- 8 years post-stroke (varied)	Cognitive scores significantly below norms; worst in perinatal group and combined lesions; different periods of vulnerability by lesion location	Unequal group sizes by age and lesion location<br>Lack of control group<br>Longer follow-up in perinatal group<br>Cognitive assessment not comprehensive
Allman C & Scott RB (2013) (36)	N: 44 children<br>Type: AIS<br>Age: 9.2 ± 3.4 years<br>Controls: none	1 month-16 years	General neuropsychological functions	3 months-11 years 5 months post-stroke	Worse neuropsychological outcomes compared to normative sample; better outcomes if stroke occurred between 1-6 years; left hemisphere lesions associated with poorer outcomes	Small sample size<br>Retrospective design<br>Lack of control group

Pavlovic J et al., 2006 (30)	N: 33 children<br>Type: AIS (neonatal and childhood)<br>Age: Childhood 2.1–18.2 years; Neonatal 1.0–3.7 years<br>Controls: none	Neonates; 2 months- 4 years 11 months; 5- 9 years 11 months; 10 years and older	IQ, processing speed, auditory short-term memory, school performance, neurological status	18-24 months post-stroke	Most children had neuropsychological problems; mean IQ was below average; performance IQ was lower than verbal IQ; younger age and epilepsy predicted worse outcome; no clear effect of lesion location was found	Small sample size<br>Lack of control group<br>Wide age range<br>Different neuropsychological tests used<br>Small subgroup sizes<br>Short follow-up period
Westmacott R et al., 2009 (37)	N: 26 patients<br>Type: Unilateral neonatal AIS<br>Age: 3 – 12 years<br>Controls: none	Neonatal (0-28 days)	IQ, Working Memory, Processing Speed, Nonverbal reasoning	Longitudinal: preschool & school-age (6+ years)	No early deficits at preschool; significant decline in Full Scale IQ, Working Memory, and Processing Speed at school age; many showed decline in at least one IQ index; no lesion laterality effect; worse outcomes observed at school age	Small sample size<br>Lack of control group<br>Different cognitive tests used at different times<br>No long-term follow-up beyond school age
Anderson V et al., 2020 (33)	N: 61 patients<br>Type: AIS<br>Age: 0 – 18 years<br>Controls: none	43.9 ±5 9.3 months	Cognitive, language, motor, adaptive, and social abilities	Baseline, 1, 6, 12 months post-stroke	Most children showed resilience at 12 months. Better outcomes were associated with older age at stroke, intact early status, and stronger language and adaptive skills. Worse outcomes occurred with cortical damage, large infarcts, neonatal onset, and MCA strokes. Seizures were also related to worse cognitive outcomes	Lack of control group<br>Follow-up only 12 months

Ledochowski J et al., 2024 (38)	N: 34 patients<br>Type: AIS (cortical, subcortical, cortical + subcortical)<br>Age: 1 month – 15 years<br>Controls: none	Childhood	Intellectual abilities, verbal reasoning, perceptual reasoning, processing speed	Early recovery (30 days-1 year) post-stroke	Early motor functioning linked to intellectual, verbal, perceptual reasoning, and processing speed; later motor functioning mainly associated with processing speed; subcortical lesions associated with milder deficits, cortical+subcortical lesions with more severe deficits	Small sample size<br>Lack of a control group<br>Retrospective design
Yvon E et al., 2018 (13)	N: 128 patients<br>Type: AIS (46) and HS (82)<br>Age: 1 month – 15 years<br>Controls: none	1 month- 15 years	Intellectual abilities, developmental domains, functional and academic outcomes	Median 43 months post-stroke	Motor deficits remained at discharge. Hemorrhagic stroke was associated with better motor and functional outcomes compared to arterial ischemic stroke. Many children required special education. IQ was the strongest predictor of academic placement. Overall, stroke caused long-term motor, cognitive, and functional impairments	Retrospective study design<br>Lack of control group<br>Data collected over 18 years, leading to variability in assessments and methods<br>No formal reliability testing of extracted data
Gschaidmeier A et al., 2021 (39)	N: 23 patients<br>Type: AIS or PVI (unilateral; 8 with epilepsy)<br>Age: 8 – 26 years<br>Controls: 23 age-matched healthy controls	Perinatal-neonatal	Non-verbal intelligence	≥ 8 years post-stroke	Patients without epilepsy performed within normal range; patients with epilepsy had impaired non-verbal intelligence; lesion size, lesion side, language lateralization, and motor impairment did not affect non-verbal intelligence;	Wide age range<br>Small sample size<br>Excluding patients with IQ < 70

					epilepsy was the only significant risk factor	
van Buuren LM et al., 2013 (40)	N: 50 patients<br>Type: PVHI (21), PAIS (29)<br>Age: 6 – 20 years<br>Controls: none	Perinatal	Intelligence, verbal memory, visual-motor integration, language comprehension, attention, concentration, reaction time, executive function	Cognitive testing began around 24 months after birth and continued at school age	Children with PVHI showed lower cognitive performance overall, especially in processing speed and visual-motor integration, while children with PAIS had deficits linked to basal ganglia and thalamic involvement; postneonatal epilepsy was associated with worse cognitive outcomes	Different tests were used across time<br>Lack of control group<br>Sample size, although relatively large<br>Wide age range
Talib TL et al., 2008 (41)	N: 5 twins<br>Type: uMCA PS<br>Age: 5 – 8 years<br>Controls: unaffected co-twins	Perinatal	IQ, verbal and non-verbal intelligence, memory, language, attention, executive function, visual-motor integration, fine motor skills	Median age 5 years at testing	Affected twins had significantly lower full-scale, verbal, and non-verbal IQ than co-twins; deficits also in verbal memory, receptive language, verbal fluency, and visual attention; large infarcts and epilepsy contributed to poorer outcomes	Small sample size<br>Some cases may have been missed during subject identification
Chea RE et al., 2019 (43)	N: 11 children<br>Type: ABI (including stroke)<br>Age: 7 – 11 years<br>Controls: 11 age-matched typically developing children	More than 12 months post-injury	Vocabulary (tiered word knowledge: basic, high frequency, curriculum-based); naming errors	≥ 12 months post-injury	Children with acquired brain injury showed poorer overall vocabulary scores than controls; accuracy similar for basic and high-frequency receptive words, but significantly lower for high-frequency expressive and curriculum-based words; most naming errors were semantic	Small sample size<br>Differences in recruitment strategies<br>Sample mostly included children with severe ABI; findings may not apply to mild/moderate cases.

Heimgärtner M et al., 2024 (44)	N: 9 children<br>Type: Unilateral stroke; 23 unilateral perinatal stroke<br>Age: 8 – 27 years<br>Controls: age-matched typically developing children	Childhood stroke: 2 months -16 years<br>Perinatal: <29 days	Language outcomes, non-verbal intelligence, lesion volume, language lateralization	≥ 1 year after the injury	All children who experienced a stroke during childhood recovered from initial aphasia; however, their language scores remained lower than those of controls, similar to children with perinatal stroke. Lesion side, lesion volume, age at stroke, and language lateralization did not significantly affect outcomes.	Small sample size<br>Different language tests
Chapman SB et al., 2003 (45)	N: 17 children<br>Type: Non-recurrent, non-progressive focal stroke lesions<br>Age: 8 – 19 years<br>Controls: 17 age- and sex-matched orthopedic controls	Stroke: 0-13 years; subdivided in <1years (n: 9), >1years (n: 8)	Discourse, macro-level story interpretation, episodic memory	≥ 1 year after stroke	Children with stroke performed worse than controls on discourse; early-injury group (<1 year) had poorer outcomes; lesion site and size did not significantly affect performance	Small sample size<br>Heterogeneous group with wide age range<br>Use of different or non-standardized assessment tools.<br>Limited long-term follow-up for assessing outcomes and interventions.
Newport EL et al., 2022 (47)	N: 15 patients<br>Type: PAIS (left hemisphere; language regions)<br>Age: 9.7 – 26.5 years<br>Controls: healthy siblings	Perinatal	Sentence processing, vocal emotion, cognitive abilities	Long-term (adolescence / adulthood)	Despite left hemisphere damage, participants developed normal sentence and vocal emotion processing in righ hemisphere; nonlinguistic cognitive impairments present; shows high developmental plasticity for language	Small sample size<br>Different language tests

Reilly JS et al., 2013 (48)	N: 35 children<br>Type: UPS (right vs. left hemisphere)<br>Age: 7.1 – 16.7 years<br>Controls: 60 healthy controls	Perinatal	Narrative language, morphosyntax, syntactic complexity	≥ 7 years post-stroke	Left hemisphere stroke shows more morphological errors, less complex syntax, and poorer story settings.<br>Right hemisphere stroke is similar to controls except for reduced syntax complexity.<br>Language plasticity persists, but left hemisphere lesions lead to deficits	Small sample size<br>Wide age range<br>Partial availability of standardized language data<br>Focus mainly on narrative language tasks
François C et al., 2016 (50)	N: 1 child<br>Type: Left perinatal stroke (MCA, supramarginal, superior parietal, insular, pre/postcentral gyri)<br>Age: 3.5 years<br>Controls: age-matched controls for word learning		Language, word learning, phonological production, linguistic complexity, cognitive and motor development	25 and 42 months post-stroke	Average cognitive, motor, and language at 25m; delayed language at 42m with limited word production and spontaneous speech; impaired novel word learning; right-lateralized activations suggest reorganization	Single case design<br>Lack of control group
Peterson RK et al., 2021 (51)	N: 30 children<br>Type: Isolated basal ganglia AIS<br>Age: 6 – 20 years<br>Controls: none	Childhood (1 month-18 years)	Expressive/receptive language, verbal fluency, narrative discourse, pragmatic language, academics, intellectual functioning	Variable	Average intellectual functioning/verbal comprehension; higher-order language difficulties (fluency, narrative, pragmatics); infarct size associated with outcomes; standardized tests may not capture all issues	Lack of control group
Sherman V et al., 2021 (52)	N: 173 children<br>Type: AIS and CSVT (67 neonates, 106	Neonatal: mean 2.9 days; childhood: mean 6.5 years	Swallowing, oral motor, speech, language, caregiver burden	Acute hospitalization	In children with AIS, dysphagia was common in both neonatal and childhood-onset cases. Oral motor, motor speech, and language	Retrospective design<br>Lack of control group

	children)<br>Controls: none				impairments were more frequent in childhood-onset stroke, with overlapping difficulties contributing to more complex clinical profiles.	Neurological severity based on non-objective ratings for some patients<br>Long-term outcomes unknown.
Leung KI et al., 2023 (53)	N: 237 children<br>Type: AIS<br>Age: 2 months – 18 years (retrospective)<br>Controls: none	Neonatal: < 28 days; First-year: 28 days–12 months;<br>Childhood: 13 months–18 years.	Language production, comprehension, cognition, behavior	2 months-10 years post-stroke	Cognitive outcomes similar across monolingual/bilingual; monolinguals with first-year stroke worse in productive language; no detrimental effect of bilingualism; bilingualism may facilitate recovery	Small, uneven groups<br>Lack of control group<br>Retrospective data<br>Short follow-up
Kolk A et al., 2011 (7)	N: 31 children<br>Type: Ischemic/hemorrhagic stroke (21 neonatal, 10 childhood)<br>Age: Neonatal mean 6.86 ± 2.52 years; Childhood mean 8.21 ± 2.61 years<br>Controls: 31 healthy controls (mean 7.34 ± 2.49 years)	Neonatal: 0-28 days;<br>Childhood: 4-10 years	Attention, language, memory, sensorimotor, executive functions	Mean 6.86 years (neonatal), 8.21 years (childhood), Average 2.72 years post-stroke	Neuromotor impairment was observed in both neonatal and childhood stroke groups. Children showed worse attention, language, memory, and sensorimotor functions compared to controls, while executive functions were preserved. Worse outcomes were associated with left hemisphere lesions and epilepsy.	Small sample size with unequal groups<br>Wide age range

Abgottspon S et al., 2022 (54)	N: 52 patients<br>Type: AIS (neonatal and childhood)<br>Age: median 15.3 years<br>Controls: 49 healthy controls (median 13.6 years)	Neonatal: 0-28 days; early childhood: 29 days to under 6 years; late childhood: 6 to under 16 years.	Intelligence, executive functions, processing speed, memory, fluency, visual-motor	2years post-stroke	Cognitive outcomes worse vs controls; early childhood stroke had worst flexibility, processing speed, and verbal learning	Age group definitions vary across studies<br>Selection bias due to loss to follow-up, deaths, young age, and non-participation.
Fuentes A et al., 2017 (56)	N: 32 patients<br>Type: Unilateral AIS<br>Age: 9.16 ± 0.43 years<br>Controls: 32 healthy controls (8.25 ± 0.42 years)	6-14 years	Working memory	≥ 6 months post-stroke	Working memory was compromised in AIS compared to controls; many showed impairments on performance tests and parent reports; subtle deficits may go undetected, so follow-up is recommended.	Small sample size.<br>No measure of the episodic buffer component of working memory<br>Testing occurred in multiple environments<br>Overrepresentation of perinatal stroke cases
Gold JJ & Trauner DA 2014 (57)	N: 27 patients<br>Type: Unilateral perinatal stroke<br>Age: 6 – 16 years<br>Controls: 19 healthy controls (7 – 14 years)	Perinatal	Memory (verbal and non-verbal), global cognition	Age range 6–16 years, mean 10.7 ± 0.6	Perinatal stroke was associated with reduced bilateral hippocampal volume. Smaller hippocampi correlated with poorer memory—verbal on the left, non-verbal on the right. Seizures further worsened hippocampal damage and memory performance.	Small sample size<br>Age ranges not perfectly matched<br>IQ differences between groups
Salzmann S et al., 2025 (58)	N: 43 patients<br>Type: AIS<br>Age: 6 – 23 years<br>Controls: none	Childhood (28 days-16years)	Intelligence, processing speed, working memory	Median 3.52 years post-stroke	Cognitive performance was related to lesion volume—processing speed declined with larger lesions, and working memory was reduced when the left caudate was	Small sample size and clinical heterogeneity<br>Lack of control group

					affected. Long-term outcomes were associated with neurological status at discharge.	Retrospective, multi-center data with variable assessments<br>Limited cognitive domains tested
Larsen N et al., 2022 (63)	N: 83 children<br>Type: AIS (n=26), PVI (n=26)<br>Age: 6 – 19 years<br>Controls: 31 age-matched typically developing controls	Various	Intelligence, processing speed, working memory.	6-19 years post stroke	PVI group had milder impairments; AIS group worse outcomes in processing speed, executive functions, and memory	Cognitive data from parent reports (subjective)<br>No direct ADHD/executive tests in controls
Shinde K et al., 2023 (67)	N: 73 patients<br>Type: AIS (n=36), PVI (n=37)<br>Age: AIS 6.3 – 19.0 years; PVI 6.6 – 19.7 years<br>Controls: 36 healthy controls (6.5 – 19.0 years)	Perinatal stroke	Executive function, ADHD		AIS patients showed higher scores than norms for executive functions and attention deficit hyperactivity symptoms, while PVI patients scored similarly to controls. Contralesional cortex morphology differed: AIS patients had increased grey matter volume and cortical folding, whereas PVI patients showed reduced grey matter in some areas. No associations were found between these changes and motor or cognitive abilities.	Retrospective design<br>Small sample sizes per group<br>No direct cognitive testing in controls

Everts R et al., 2023 (68)	N: 25 patients<br>Type: AIS<br>Age: 5 – 16 years<br>Controls: none	5–16 years	General intelligence, Abstract reasoning, Verbal learning, Verbal memory, Verbal fluency, Processing speed, Visual-motor integration, Inhibition, Cognitive flexibility, Working memory	>2 years post-stroke	Lesion measurements obtained through different methods were closely aligned, particularly for smaller lesions. Lesion size was significantly associated with clinical outcomes and processing speed, even after accounting for age at stroke and multiple comparisons.	Small, clinically heterogeneous sample<br>Selection bias<br>Lack of control group
Li E et al., 2022 (64)	N: 18 patients<br>Type: Perinatal stroke<br>Age: 6 – 16 years<br>Controls: none	Perinatal	Executive function, math performance, visuospatial processing, IQ	Variable	Children with perinatal stroke scored below norms in executive function, math, visuospatial processing, and IQ; poorer inhibitory control linked to worse math; older age associated with better visuospatial scores; females performed better on inhibition tasks	Small sample size, with some missing data<br>Lack of control group
Meghji S et al., 2024 (65)	N: 120 children<br>Type: AIS (n=31), PVI (n=30)<br>Age: AIS 6.6 – 19.5 years; PVI 6.7 – 19.7 years<br>Controls: 59 healthy controls (6.5 – 19.0 years)	6-19 years	Executive function, ADHD symptoms	Variable	AIS and PVI groups showed lower functional connectivity within and between attention, frontoparietal, and default mode networks compared to controls. Connectivity differences were associated with parent-reported executive function deficits and ADHD symptoms.	Limited cognitive data, especially in the control group.<br>No screening for ADHD/executive dysfunction in controls; stimulant use not recorded.
O'Keeffe F et al., 2014 (70)	N: 49 children<br>Type: AIS (MCA territory, basal	4 months-15.66 years	General intellectual ability, attention, working memory,	≥ 1 year post-stroke; subgroup n=9	Focal deficits in attention (response inhibition, dual attention) and executive	Lack of control group

	ganglia often involved; unilateral/bilateral)<br>Age: 6.0 – 18.4 years<br>Controls: none		processing speed, executive function, emotional and behavioral regulation	followed 19-31 months	function beyond general IQ; emotional and behavioral dysregulation common; deficits present irrespective of hemisphere; earlier stroke associated with better executive performance supporting early plasticity hypothesis	Normative differences across cognitive tests may affect comparability.<br>Sample heterogeneity, including seizure history and disability level.
Long B et al., 2011 (61)	N: 28 patients<br>Type: IS (n=21), HS (n=7)<br>Age: 10 – 15 years<br>Controls: none	Stroke sustained > 18 months prior to testing (age at onset variable)	Executive functions (attentional control, goal setting, cognitive flexibility, information processing, everyday executive function)	Single assessment (≥ 18 months post-stroke)	Deficits in executive functions were found despite overall normal IQ; both frontal and extra-frontal lesions similarly affected cognitive performance. Everyday executive difficulties were more pronounced with frontal lesions; subcortical frontal lesions were linked to everyday executive impairments. Findings support a diffuse representation of executive functions in the immature brain.	Small sample size<br>Lack of control group<br>Stroke etiology was heterogeneous<br>Wide age range at stroke and at assessment<br>No demographically matched control group
Long B et al., 2011 (62)	N: 28 patients<br>Type: IS (n=21), HS (n=7)<br>Age: 10 – 15 years<br>Controls: none	Stroke sustained > 18 months prior to testing (age at onset variable)	Executive functions (attentional control, goal setting, cognitive flexibility, information processing, everyday executive function)	Single assessment (≥ 18 months post-stroke)	Executive function deficits present despite normal intelligence; larger lesions linked to worse executive functions; executive functions impaired even with non-frontal lesions	Small sample size<br>Lack of control group<br>Stroke etiology was heterogeneous<br>Wide age range at stroke and at assessment

						No demographically matched control group
Kollndorfer K et al., 2025 (72)	N: 14 patients<br>Type: AIS<br>Age: 12.7 ± 3.12 years<br>Controls: 14 age- and sex-matched controls	Stroke age: 1 month to 16 years	Executive functions: cognitive flexibility, working memory, planning, sustained attention	Variable	Patients showed weaker executive function compared to controls, though most scores remained within the normal range. Lesion size was linked to difficulties in sustained attention and some aspects of daily executive functioning. However, when accounting for sustained attention, the link between lesion size and executive difficulties disappeared, suggesting that sustained attention mediates this relationship.	Small sample size<br>Patient heterogeneity in age at stroke and assessment.<br>Strict inclusion criteria
Everts R et al., 2010 (74)	N: 10 children<br>Type: Unilateral ischemic stroke<br>Age: 10 – 19 years<br>Controls: 20 healthy right-handed controls (8 – 20 years)	0 months 3 days-<br>15 years 4 months (mean 8 years 1 month)	Language (productive & semantic), visual search (simple & complex)	Mean 6 years 4 months post-stroke (range 1 year 8 months - 11 years 7 months)	Atypical lateralization occurs frequently in visual search, predominantly left-lateralized, and less commonly in language, with some right-lateralization. Functional dissociation between language and visual search tasks is observed, involving contralateral activations in regions typically showing developmental decreases. No significant differences in task performance were found compared to controls.	Small sample size<br>Analyses could not fully account for differences in lesion location, size, timing, or nature.<br>No longitudinal data to distinguish brain development from reorganization after stroke.

Nenning KH et al., 2025 (75)	N: 16 patients<br>Type: AIS (single focal lesions)<br>Age: 13.1 ± 2.10 years<br>Controls: 17 healthy age- and sex-matched controls	Mean 9.7 years	Executive functions, fluid reasoning, working memory	≥ 3 months post-stroke (mean interval varies)	Stroke patients showed reduced fluid reasoning and working memory scores vs controls. Altered resting-state fMRI dynamics: increased frontoparietal network state, decreased default mode network deactivation. Lesion location impacted functional brain dynamics and cognitive outcomes more than lesion size alone.	Small sample size<br>Heterogeneous ages at stroke and examination with variable time since stroke<br>No maximum time limit between stroke and assessment
Christerson S & Strömberg B 2010 (76)	N: 46 patients<br>Type: AIS (n=26), HS (n=21), SVT (n=4)<br>Age: 5.5 – 26.1 years<br>Controls: none	Age at first stroke: from 28 days to 18 years	Neurological status, school performance, quality of life, daily activities	1.6-8.6 years (median 4.2 years) after first stroke	Most had some acquired long-term deficits, including neurological impairments and school performance difficulties. No significant differences were found by stroke type, sex, or age at stroke. Outcomes ranged widely from severe deficits to full recovery.	Small, heterogeneous sample<br>Missing data<br>Lack of control group<br>New tool used lacks strong validation
Deotto et al., 2019 (78)	N: 32 patients<br>Type: Unilateral AIS<br>Age: 9.5 ± 2.7 years<br>Controls: 32 healthy matched controls	Stroke before 14 years; at least 6 months post-stroke at testing	Executive functioning (esp. metacognition), math, spelling, IQ	≥ 6 months post-stroke	Children with stroke showed significantly lower scores in math, spelling, and metacognition. Math was particularly impaired. Metacognition was a strong predictor of academic deficits. Cortical and subcortical lesions, as well as later age at stroke, were associated with worse outcomes. All patients	Small sample size<br>Limited age range<br>Overrepresentation of perinatal and cortical strokes.<br>Potential bias in parent reports and testing conditions.

					attended mainstream education; over half had IEPs.	
Greenham M. et al., 2017 (89)	N: 31 patients<br>Type: AIS<br>Age: 3.8 – 15.4 years<br>Controls: none	0-16 years	General cognitive function including reasoning, memory, and learning abilities; assessed using age-appropriate standardized tests measuring overall intelligence and cognitive development at 12 months post-stroke.	5 years post-stroke (predictors at 12 months)	Poorer cognitive function at 12 months post-stroke predicted significantly worse psychological and social outcomes at 5 years. Larger lesion size and poorer early psychological functioning also predicted increased behavioral problems. Neurological status and early social competence were not significant predictors.	Small sample size<br><br>Lack pof control group<br><br>Mostly young children<br><br>Parent ratings only; lacks teacher/self perspectives.

AIS: Arterial Ischemic Stroke; HS: Hemorrhagic Stroke; PVI: Periventricular Infarction; PVHI: Periventricular Hemorrhagic Infarction; uMCA PS: Unilateral Middle Cerebral Artery Perinatal Stroke; ABI: Acquired Brain Injury; IS: Ischemic Stroke; SVT: Sinovenous Thrombosis.
